# A Critical Review of the Crucial Role of the Yellow River’s Sediment in the Interfacial Migration and Fate of Pollutants and Prospects for the Application of Environmental Sediment Restoration

**DOI:** 10.3390/toxics12090669

**Published:** 2024-09-14

**Authors:** Xiaojuan Sun, Zhenzhen Yu, Qiting Zuo, Quantao Cui, Ziyu Song, Lin Gong, Shoushu Liu, Wei Zhang

**Affiliations:** 1 School of Water Conservancy and Transportation, Zhengzhou University, 100 Kexue Avenue, Zhengzhou 450001, China; sunxiaojuan@gs.zzu.edu.cn; 2 Yellow River Water Resources Protection Institute, No. 12 East Chengbei Road, Zhengzhou 450004, China; zzyuhydro@126.com; 3 Henan International Joint Laboratory of Water Cycle Simulation and Environmental Protection, Zhengzhou 450001, China; 4 Zhengzhou Key Laboratory of Water Resource and Environment, Zhengzhou 450001, China; 5 Yellow River Institute for Ecological Protection and Regional Coordination Development, Zhengzhou University, 100 Kexue Avenue, Zhengzhou 450001, China; 6 School of Ecology and Environment, Zhengzhou University, 100 Kexue Avenue, Zhengzhou 450001, China; cuiqta@163.com (Q.C.); songziyu@stu.zzu.edu.cn (Z.S.); lingo716@163.com (L.G.); lss15036301078@163.com (S.L.)

**Keywords:** Yellow River sediment, interfacial migration, pollutants, future prospects, environmental restoration

## Abstract

Considering the increasing sediment content and increasing sediment flux of the Yellow River over the years, it is of significance to investigate the potential interfacial force mechanism between pollutants and Yellow River sediment. This article has reviewed the current research on the Yellow River sediments’ mineral structures while investigating the potential interaction force between sediment and pollutants in the water environment. This article has conducted a comprehensive analysis of the influence of sediment on the migration of pollutants in the water environment. What is more, the authors have provided an outlook on the future applications of sediment in ecological environmental systems. Yellow River sediment mainly included minerals and some clay phases, while its irregular surface provided sites for the interface adsorption of pollutants. The interface force between the sediment and pollutants is mainly attributed to promoting bacterial growth on the surface of sediments, physisorption, and chemisorption forces. The sediments carry and transport pollutants during the long-distance water flow migration process. The sediment should be effectively utilized and better integrated into ecological or environmental restoration systems. This article provides a reference for studying the behavior of Yellow River sediment and the direction of future efficient utilization.

## 1. Introduction

The Yellow River, the fifth-largest river in the world, supplies important water resources for approximately 12% of China’s population and 15% of its agricultural irrigation [1], holding a core position in China’s economic and social development and ecological security. The important instructions of ecological environment protection and high-quality development have already been addressed to the Yellow River basin, elevating it to a national strategic position, further emphasizing the crucial role of the Yellow River in China’s economic and social development.

Since the 1980s, domestic economic construction has been advancing comprehensively, and the contradiction between the supply and demand of water resources in the Yellow River has been increasingly acute [2,3]. Thus, the water quality in key sections and main tributaries of the Yellow River has generally exceeded water quality standards. Based on the Environmental Quality Standards for Surface Water of the People’s Republic of China [4], the classification of water quality into Classes I to V is due to various indicators in the water, including dissolved oxygen, metal elements, microorganisms, and chemical substances. According to the monitoring data, in the 1990s, Class I~III water quality sections accounted for 65.8% of the Yellow River basin, while the sections with inferior water quality accounted for 34.2%, including 4.9% of Class V sections. In recent years, with the increased attention and stronger governance by the state, the water quality of the Yellow River basin has obviously improved. In February 2023, the Ministry of Ecology and Environment announced that the main stream of the Yellow River had already reached Class II water quality level throughout its entire length in 2022. The continuous improvement of the water quality of the Yellow River contributes to the development of environmental management and related policies in China. Additionally, the interaction between pollutants and sediments also further promotes the future management of the Yellow River.

Although significant progress has been made in the protection of the water ecological environment, there are still many prominent problems and shortcomings to be urgently addressed. The current water quality assessment system has not included emerging pollutants (such as microplastics, polycyclic aromatic hydrocarbons, antibiotics, and PFAS pollutants, etc.), that have already been detected in partial sections of the Yellow River basin [5,6,7,8]. River sediments are gradually coming to serve as primary sinks and reservoirs for contaminants in the aquatic environment [9]. Thus, in the Yellow River, the combination of traditional pollutants and emerging pollutants in the water or sediment phase would inevitably increase the pollution status and ecological risks of the basin’s water environment. However, comprehensive studies on emerging pollutants and their behavior in sediment-rich environments are deficient, leading to ineffective control of emerging pollutants in multi-sediment rivers. Considering the complex molecular and microstructural characteristics of the complex pollutants (traditional pollutants and emerging pollutants), the diverse and unpredictable interface of physicochemical interactions between river particles and pollutants are probably present [10]. Especially in the case of increasing sediment content and flux in the Yellow River over the years [11,12], it is important to investigate the coexistence and migration behavior of multiple pollutants with the existence of sediment to predict the environmental application potential of sediment interfaces in removing pollutants from the water environment [13].

The objective of this review was to emphasize the crucial role of sediment (including suspended sediment and deposited sediment) in the interfacial interaction and migration of pollutants. Firstly, the properties of sediment were systematically reviewed and analyzed to explore the fundamental micro-interface structure and size distribution of sediment in the river environment. Subsequently, we investigated the potential interaction mechanism between the sediment and pollutants at the microscale, further revealing the influence mechanism of sediment on the transportation and dispersion process of pollutants in the river. Based on the progress of the role of sediment in carrying pollutants, the future perspectives of higher value-added application of sediment in the area of environmental pollution are proposed in this article. Previously, there had been no literature reviewing the application of Yellow River sediment in pollutant treatment, making this a novel and significant review. Additionally, unlike other river basins, the Yellow River basin has the highest possible sediment content, and reviewing its application in environmental remediation is of great importance.

We searched approximately 300 papers in the Web of Science database, primarily using keywords such as “Yellow River sediment”, “interfacial migration”, “pollutants”, “future prospects”, and “environmental restoration”. Ultimately, we selected around 60 papers for summary and analysis.

## 2. Yellow River Sediment Composition and Characteristics

**Figure 1:**
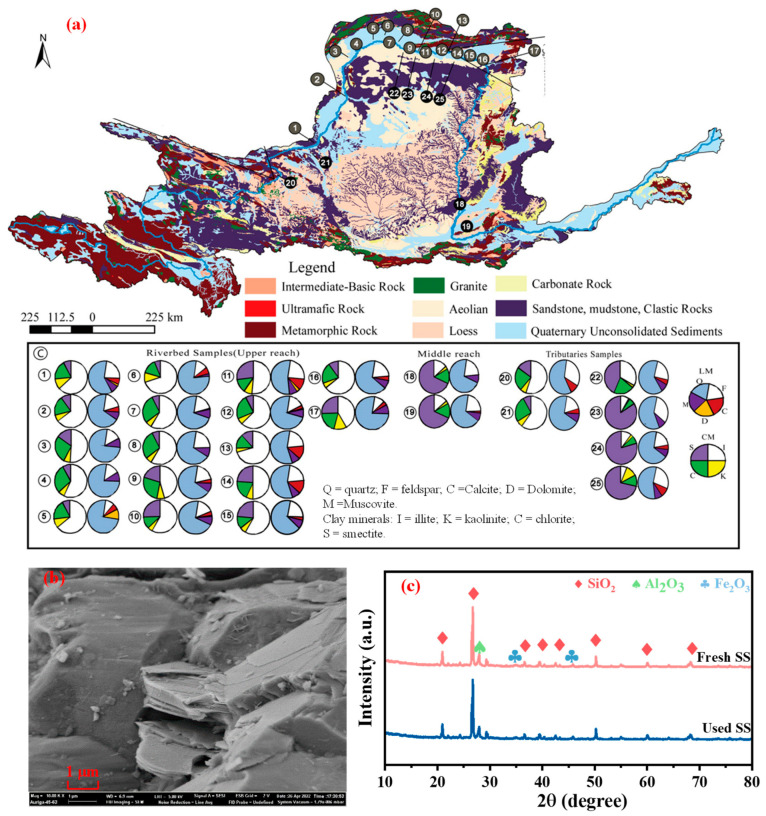
(**a**) The main geological conditions along the Yellow River and the dominant mineral composition in the collected sediment samples; (**b**) SEM image of the collected suspended sediments downstream of the Yellow River [14,22]; and (**c**) XRD patterns of the collected suspended sediments downstream of the Yellow River [14,22].

More attention was paid to the structural characteristics of sediments [14,15,16], due to the higher content of sediment in the Yellow River when compared with other rivers around the world. In 2021, the average sediment concentration of the Yellow River basin reached 4.33 kg/m^3^, significantly higher than the average sediment concentrations of other rivers [17]. Generally, the composition of sediment mainly includes quartz (Q), feldspar (F), calcite (C), dolomite (D), muscovite (M), and some clay minerals (illite, kaolinite, chlorite, and smectite). In addition to the components mentioned above, some trace heavy metals (including Fe, Mn, Pb, Cu, Zn, Cr, Ni, and Al, etc.) were also distributed on the surface of the sediment through chemical bonding or physical adsorption processes [18], while concentrations of trace heavy metals were determined by a flame atomic absorption spectrophotometer. Therein, the core chemical components were silicates and silica phases (Figure 1), while a few magnetic components (such as limonite, magnetite, ilmenite, and anatase [19,20]) could also be found in the structure of the sediment. Those above-mentioned magnetic minerals would have an interface adsorption effect on pollutants in the river environment [14,21]. In terms of the microscale morphology of sediment, the Yellow River sediments usually exhibited an irregular surface with certain concavities and convexities, containing irregular pores [14]. Table 1 comprehensively summarizes the basic properties of the sediment collected from locations along the upper, middle, and lower reaches of the Yellow River at different periods.

As shown in Table 1, many scholars have already analyzed the sediment samples collected from the Yellow River and demonstrated that the size and density of sediment collected from different sites have certain differences [23,24,25,26,27]. Overall, from the aspects of the sediments’ morphology, the sediments collected from the Yellow River mainly consisted of clay, silt, and sand, among others. The sizes of Yellow River sediments varied significantly and fluctuated within a certain range, which was mainly related to the erosion and fragmentation that would cause a reduction in the size of sediments in the water [23,24,26,27]. The surfaces of these structures, which contain pores, could provide interface sites for the adsorption of pollutants from the water environment [14].

## 3. Interaction Mechanisms of Pollutants with Sediment

### 3.1. The Interface Interactions between Sediment and Pollutants at the Microscale

**Figure 2:**
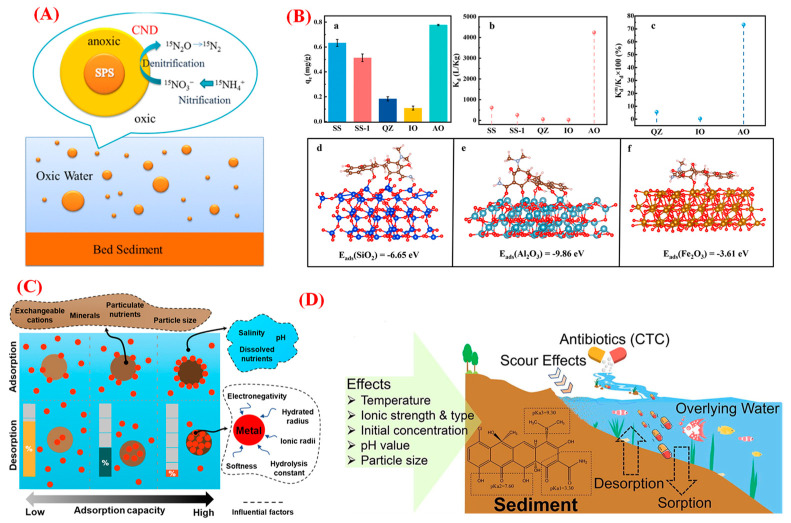
The potential interface interactions between the sediment and pollutants: (**A**) sediment acting in the nitrification/denitrification process. (**B**) contributions of different components of sediment to the adsorption of TC. Herein, (a) presenting adsorption capacity of different components of SS to TC; (b) K_d_ values and (c) presenting the contributions of different mineral fractions to the overall adsorption coefficients; adsorption energies (d) SiO_2_, (e) Al_2_O_3_, and (f) Fe_2_O_3_ for TC adsorption. (**C**) sediment interfacial reaction with heavy metal ions; (**D**) potential interfacial reaction between sediment (collected from the Lanzhou section) and CTC.

The interactions between the Yellow River sediment surface and pollutants are complex on the microscale [2,28]. The scholars of Xia et al. (2017) have indicated that the interface of suspended sediments would influence the process of nitrification/denitrification (Figure 2A), while the rates of the nitrification/denitrification process would be significantly impacted by the size of sediment particles. Therein, the suspended sediments with smaller particles would usually have a larger specific surface area and higher organic content, promoting bacterial growth and subsequently increasing the nitrification/denitrification rate [15]. In the water, the bacteria would tend to attach to the surface of suspended sediments, while the maximum growth rate of bacteria at the suspended sediment–water interface was around twice that in the water phase [16]. What is more, the presence of low-oxygen microsites in the suspended sediments would be beneficial to the denitrification process [29]. This means that the surface of suspended sediment could efficiently provide interface sites for the growth and activities of microorganisms in the water environment. Previous research has demonstrated that a total of 22 antibiotics were detected, with maximum concentrations ranging from 0.27 to 30.14 ng/L in the main stream of the Yellow River [30]. Cui et al. studied the potential interface contact between the suspended sediment and tetracycline (TC, a typical emerging pollutant) in the water environment, proving that the physisorption and chemisorption forces would contribute to TC adsorption by suspended sediment (Figure 2B). Interestingly, the theoretical calculation results demonstrated that the main mineral components of Al_2_O_3_ (of suspended sediment) contributed most to the TC adsorption process, compared with the other main components (mainly SiO_2_ and Fe_2_O_3_) of the suspended sediment [14]. These results indicated that the larger proportion of the main components in the SS would not necessarily lead to a greater contribution to TC adsorption by suspended sediment in the water environment. Commonly, a decrease in the particle size of sediments would thereby increase their specific surface area, resulting in a significant improvement in their adsorption efficiency for some pollutants (for example, polycyclic aromatic hydrocarbons, PAHs) [31]. Heavy metals have accumulated in the surface sediment, causing serious ecological security risks. Research has indicated that the concentration ranges of the heavy metals in the sediment of the Yellow River were: Zn, 41.8–114 mg/kg; Pb, 4.27–42.5 mg/kg; Ni, 15.0–39.6 mg/kg; Cu, 6.99–261 mg/kg; Cr, 42.6–132 mg/kg; Cd, ND-0.252 mg/kg; and As, ND-8.67 mg/kg, respectively [32]. The selective adsorption capacity of heavy metal ions by sediment particles was closely related to the softness of heavy metal ions (Figure 2C), showing the sequence Cu > Pb > Ni > Zn [33], while the particle sizes of the sediment and the existence of particulate nutrients and exchangeable cations would significantly affect the adsorption of heavy metals by sediment in the river environment. However, the heavy pollutants adsorbed on the surface of the sediment would also be released into the water environment through the desorption process. The scholars Liang et al. systematically analyzed the adsorption–desorption interface of CTC (chlortetracycline) by sediment collected in the Lanzhou section of the upper reaches of the Yellow River (Figure 2D), demonstrating that factors such as the content of clay and organic matter, as well as CEC (cation exchange capacity) would affect the adsorption and desorption behavior of CTC by the sediment [34]. As shown in Table 2, the pH value, ion types, and strength of the water environment would also significantly affect the interface adsorption process of CTC by the sediment [34]. What is more, the sediments in other rivers, for example, the Bahe River (the largest river in the city of Xi’an, China), would also adsorb the emerging pollutants of phenol and bisphenol A from the water environment [35]. Therein, only the humic acid (HA) concentration would obviously affect the adsorption process of phenol by the sediment. However, the influence of environmental factors on the adsorption of pollutants by sediment are ranked in the following order: the particle size of the sediment > HA concentration > pH > contact temperature. Considering the above analysis, from the microscopic perspective, the varying environmental factors would create significant differences in the adsorption of pollutants by the sediment.

### 3.2. Transport Pathways and Dispersion of Pollutants with the Influence of SS

Generally, in the river, the suspended sediment or particulate matter tends to adsorb pollutants. The adsorption of pollutants by suspended sediments is influenced by several factors such as the size and chemical composition of the particles, the type of pollutants, and interactions between the particles and pollutants. Even during long-distance transportation, the pollutants were still attached to the sediment, posing potential hazards to the ecological environment system [10,37,38]. However, when the river’s natural environment changed, the pollutants could be desorbed from the sediment [39]. During the water flow migration process, the smaller sediment particles usually carry increasing amounts of pollutants due to their larger relative surface area [40].

**Figure 3:**
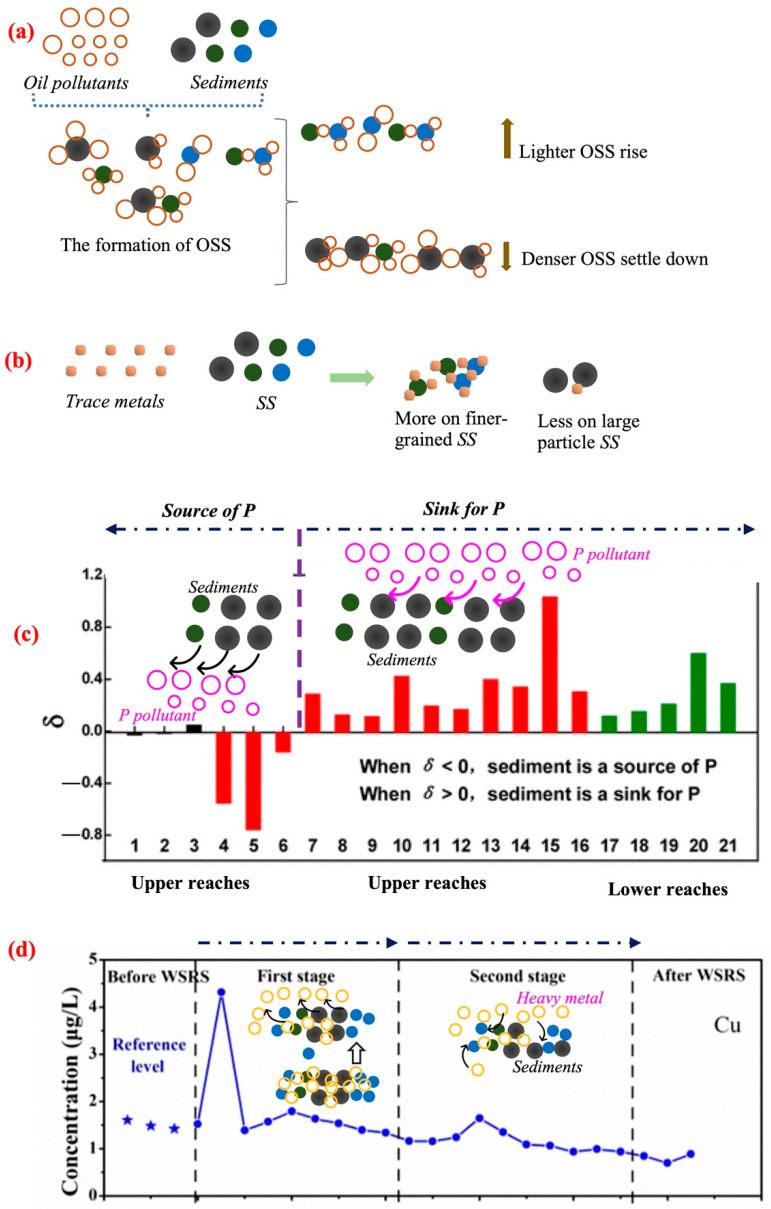
The potential influence of SS on transport pathways and dispersion of pollutants: (**a**) SS with oil pollutants; (**b**) SS with trace metals; (**c**) SS with P; (**d**) SS with heavy metals.

Commonly, the suspended sediment was critical in the transportation of PAHs (polycyclic aromatic hydrocarbons) in the water environment. The PAHs were adsorbed on the surface of suspended particulates and diffused into different water environments as the particulates migrated. Therein, the suspended sediments mainly interface with PAHs with 2–3 rings, while the deposited sediments predominantly carried the 4–6 rings PAHs; this can probably be attributed to the varying densities of PAHs with different numbers of rings [41]. The water and sediment regulation of the Xiaolangdi Reservoir was capable of causing large-scale changes in the concentration of PAHs. For example, the total dissolved concentration of PAHs was 149 ± 56.3 ng/L before water and sediment regulation, and it increased to 256 ± 32.6 ng/L during water regulation and to 608 ± 69.7ng/L during sediment regulation [42]. As shown in Figure 3a, the polar molecular components of oil pollutants would interfacially interact with the SS in the water environment, thereby forming OSSs (Oil–SS aggregates) [43]. Therein, under the influence of water flow disturbance, the denser and lighter OSSs would selectively enter the deposited sediment section and SS section, respectively [40]. What is more, the SS could also carry trace metal elements, while this process was mainly controlled by the grain size of SSs [39,44]. Compared with large particle SSs, the finer-grained SSs would usually carry more trace metal elements in the water environment [45] (Figure 3b).

In the Yellow River, the SSs could serve as sources and sinks of P (phosphorus) pollutants (Figure 3c). In the upper reaches of the Yellow River, a significant portion of SS originated from the erosion of soil, containing a higher concentration of total P. Consequently, the SSs in the upper reaches of the Yellow River could be acting as the source of P. However, in the middle and lower reaches, where abundant SS exists, the interfacial adsorption between SSs and P played a dominant role in the removal of P from the water environment. Herein, the SSs were acting as sinks for P, facilitating the transfer of P from the water phase to the SS phase [46].

Aiming to maintain the ecological balance of the Yellow River basin, protect downstream areas from floods and desertification, and efficiently allocate the water resources in different regions, the water and sediment regulation (WSR) process was conducted annually in the vicinity of the Xiaolangdi (XLD) Reservoir in the Yellow River basin. During the WSR process of the XLD Reservoir of the Yellow River, the concentration of dissolved heavy metals in the water initially increased during the first stage, which was attributed to the release of heavy metals from suspended sediment particles under the water flow. Subsequently, the heavy metals in the water were further adsorbed by the SS, resulting in a decrease in the concentration of heavy metals in the Yellow River [47,48]; the mechanism flowchart of this process is shown in Figure 3d. Compared with the dissolved heavy metals, the particulate heavy metals were the dominant phase when the heavy metals transferred from the XLD Reservoir to the sea [49], which was attributed to the adsorption of heavy metals by SSs in higher concentrations. Therefore, its essence lies in the interface adsorption of pollutants by higher concentration SSs, driving the temporary or permanent association between pollutants and SSs. Further, according to previous studies by scholars, the water and sediment regulation process would influence the migration and concentration of heavy metal in the Yellow River by changing the SS transport mode [50], the transformation of SS characteristics [49], and hydrological processes [49], etc. In addition to heavy metals, the water and sediment regulation process of the Yellow River would also affect the migration behavior of other pollutants, including POC (particulate organic carbon) [51], radioactive pollutant of uranium [52], dissolved organic carbon (DOC) [53], etc. As shown in Table 3, more scholars were inclined to investigate the migration of HMs during the WSR process, due to the higher ecological risks and longer-term stability of HMs. In summary, during the WSR process, the frequent and strong hydraulic force facilitated the release and redistribution of pollutants in the Yellow River.

## 4. Prospect for Sediment-Based Restoration Route

**Figure 4:**
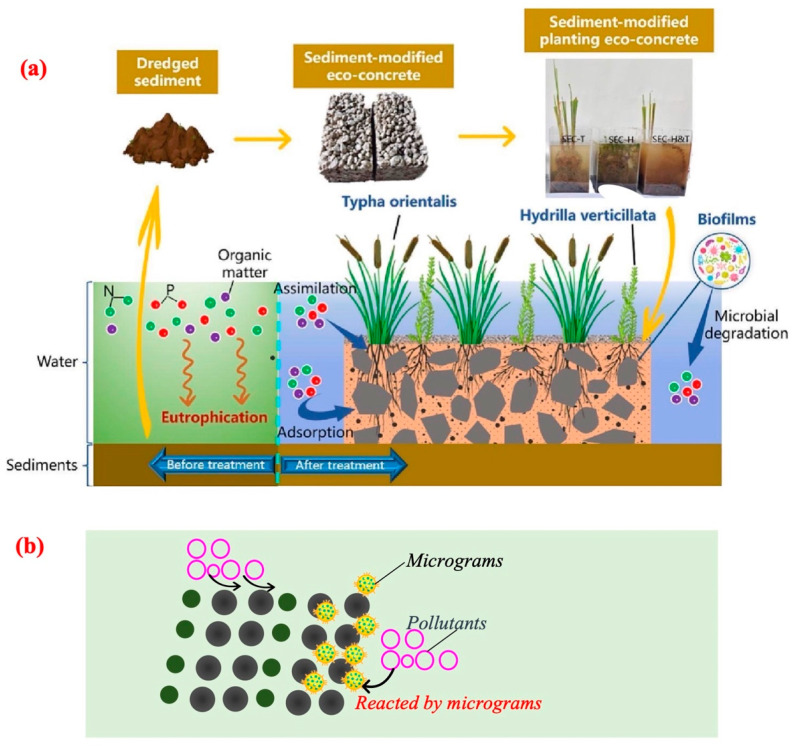
Potential application of sediment in the environmental restoration process: (**a**) sediment application in eco-concrete materials; (**b**) a mechanism diagram of sediment acting in the environmental remediation process.

Due to their unique interfacial microstructure and porosity, sediments inevitably interact with pollutants (including traditional pollutants and emerging contaminants) [56,57]. Therein, they could be considered for application in the remediation of environmental water pollution or similar fields [58]. Some scholars have introduced sediments into the construction of eco-concrete materials that were used in riverbank restoration projects. The eco-concrete materials with the introduction of sediments meet basic requirements such as compressive strength. Furthermore, introducing sediments into the eco-concrete materials (equipped with plants) could lead to significant reductions in the COD, TN, and TP by 58.59%, 74.00%, and 79.98%, respectively [56]. Therein, the sediment of the eco-concrete system could efficiently promote the survival of beneficial microorganisms in the water environment, while the adsorption of pollutants by the sediments could also effectively prevent the release of pollutants into the water (Figure 4a). Furthermore, the sediment can also be used to replace non-renewable resources for construction. For instance, sediment has been reused as the foundation or base layers in road engineering [59]. For the manufacture of mortar, sediment can replace sand as a conventional aggregate. Sediments have also been recycled in brick production, artificial aggregate, and urban landscaping works [60]. The application of sediments as soil amendment materials for plant cultivation in agriculture is also a promising means of sediment recycling. Sediments provide abundant nutrients such as N and P to crop production, reducing the application of non-renewable mineral P and N fertilizers [61]. Thus, sediment could significantly improve the removal performance of pollutants from the water while simultaneously maintaining the stability of the ecological system.

Therefore, it is of great significance to effectively utilize the sediment (suspended or deposited) with micro-interface properties, considering its basic characteristics for the adsorption of pollutants. Further, the sediment could provide microbial attachment sites and structural stability, thereby better integrating sediment into the ecological restoration system (Figure 4b).

## 5. Conclusions

This article systematically reviewed the current research on the mineral structures of Yellow River sediments, analyzing the interaction processes between sediment and pollutants in the water environment from microscale aspects, and further exploring the driving forces of interactions between sediment and pollutants. In addition, this article also conducted a comprehensive analysis of the influence of sediment on the migration of pollutants in the water environment. Furthermore, an outlook on the future applications of sediment was provided for sediment recycling in ecological environmental systems. Overall, our overview offers a comprehensive understanding of the function of sediments in the environmental field, underscoring the potential utilization of Yellow River sediments in the future.

(1) The Yellow River sediment mainly includes quartz, feldspar, calcite, dolomite, muscovite, and some clay minerals. The sediment commonly exhibits an irregular surface with certain concavities and convexities, providing certain sites for the interface adsorption of pollutants in the water environment.
(2) From the perspective of microstructure, the sediments have various forms of interface interactions with pollutants, including promoting bacterial growth on the surface of sediments, physisorption and chemisorption forces, etc. The natural water environment would inevitably affect the interface force between the sediment and pollutants.
(3) During the long-distance water flow migration process, sediments commonly carry and transport pollutants, which is mainly affected by the grain size of the sediment. During the WSR process, interface reactions between sediment and pollutants involve many subprocesses: the pollutants are generally first released from sediment and then absorbed on the sediment surface at the following stage.
(4) We should effectively utilize sediments, as they could provide microbial attachment sites and structural stability, thereby integrating sediment into ecological or environmental restoration systems.

## Figures and Tables

**Table 1:** The overview of basic properties of sediment collected from the Yellow River.

Sampling Locations	Size of Collected Samples (μm)	The Main Composition	Bulk Density (g/cm^3^)	Ref.
>Station Lijin	(1) Clay and very fine silt (<8 μm): 36~67% (2) Sand (>63 μm): 0.9~20% (3) Fine silt (8–16 μm): 17~32% (4) Medium silt (16–32 μm): 10~30% (5) Coarse silt (32–63 μm): 7~24%	Clay and very fine silt	/	[23]
Ningxia–Inner Mongolia sections	(1) Sediments from the desert sections and part of the tributary sections: 63~250 μm (2) Sediments found in some parts of the desert sections: 250~500 μm	/	/	[24]
Jinzhuang diversion Canal	/	Clay: 3.8%; Silt: 18.28%; Sand: 77.92%	1.5	[25]
Lower Yellow River at Hekouzhen and Longmen; tributaries at Zhaoshiyao on the Wudinghe River and Huaxian on the Weihe River	Most more than 0.05 mm	/	/	[26]
Yellow River mouth and the nearby Bohai Sea	Mean grain size of >200 μm	/	Volume concentration of >100 μL/L	[27]
Upper and middle reaches of the Yellow River	Average grain size between 13 μm (for SS) and 223 μm (for bedload sediments)	(1) In the upper reaches and tributaries flowing through the Loess Plateau: illite (41–70%), chlorite (8–38%), smectite (2–35%), and kaolinite (5–12%); (2) In middle reaches and tributaries flowing through the Ordos Plateau: smectite (44–87%), illite (6–33%), chlorite (4–21%), and kaolinite (1–11%).	/	[22]

**Table 2:** The crucial role of sediment in removing different pollutants from the water environment.

Sampling Location	Pollutant	Dosage of Pollutant	Dosage of SS	Influence Factors	Removal Ratio (%) and Capacity (mg/g)	Ref.
Garden mouth of Yellow River	Tetracycline	2.5~25 mg/L, 25 mL	6~10 g/L	pH, temperature, ionic types, and organic matter	80.5~67.2%/ 0.32~2.63 mg/g	[14]
Longmen hydrological station of Yellow River	Nitrogenous pollutants	5 mg/L ^15^NH_4_^+^ -N	8 g/L	Particle size of sediment	^15^N_2_ emission rate: 1.15 mg-N/(m^3^·d) at particle size of below 2 μm; ^15^N_2_O emission rate: 1.05 μg-N/(m^3^·d)	[15]
Lanzhou section of Yellow River	CTC	0~30 mg/L	1.0 g	pH, ionic types & strengths, and sediment size	0.71~0.60 mg/g (pH 3.0)	[34]
Yellow River to the mouth of the river entering the Bohai	Hg and MeHg	total-Hg of 0.519 ± 0.373 ng/L; total-MeHg of 206 ± 84 pg/L	/	SPM characteristics, pH, electrical conductivity, salinity, DOC, and Cl^−^	Hg of 99.1 ± 0.74% in SPM phase; MeHg: 86.3 ± 8.46% in SPM phase	[36]
Bahe River, in the city of Xi’an, China	Phenol and bisphenol A	10 mg/L, 10 mL	1.0 g	Particle size, humic acid concentration, pH, and contact temperature	Phenol: 0.477–4.879 mg/kg, (average 1.352 mg/kg); bisphenol A: 0.082–0.221 mg/kg, (average 0.241 mg/kg)	[35]

**Table 3:** Effect of WSR process on the migration and fate of pollutants.

Year	Duration Time	Types of Pollutants	Research Area	WSR Result	Ref.
2022	/	Heavy metals (V, Cr, Ni, Co, Cu, Zn, and Cd)	Henan section of Yellow River before WSR; from XLD downstream to end of Henan Section	(1) Increased likelihood of heavy metals released from the sediments; (2) Cr was the most released	[50]
2019	Approximately 43 days	Dissolved heavy metals (Cu, Cd, Pb, Cr, As, and Ni)	XLD station and Lijin Station	(1) XLD Station: Significant influence on the concentration of Cu, Cd, Pb, Cr, and As in water; (2) Lijin Station: Heavy metals were firstly released from sediment, then adsorbed by sediment	[47]
2018	3 July to 30 July	Heavy metals (Cr, Ni, Cu, Zn, As, Pb, and Cd)	Lijin Station	(1) Heavy metal transfer was mostly affected by the hydrological process; (2) Particulate heavy metals dominated during transfer to sea	[49]
2018	3 July to 30 July	Heavy metals (Cd, Cr, Cu, Ni, Pb, and Zn)	XLD reservoir area and its downstream	HMs distributed from sediments along the riverbank to XLD downstream	[54]
2019	26 June to 8 August	POC	Near Yellow River mouth	POC concentration correlated with SS transport	[51]
2018	/	Total organic carbon (TOC), total nitrogen (TN)	XLD station and Lijin Station	TOC and TN decreased by around 40% in the channel sediment	[55]
2013	19 June to 10 July	DOC	XLD (2 km downstream), Jiaogong Bridge, Huayuankou Station, Aishan station, Luokou Station, and Lijin Station	WSR without obvious impact on DOC	[53]

## Data Availability

Data are contained within the article.
